# Methicillin-Resistant *Staphylococcus aureus* (MRSA): Resistance, Prevalence, and Coping Strategies

**DOI:** 10.3390/antibiotics14080771

**Published:** 2025-07-30

**Authors:** Jiajing Li, Fusheng Cheng, Xiaojuan Wei, Yubin Bai, Qing Wang, Bing Li, Yaxin Zhou, Bintao Zhai, Xuzheng Zhou, Weiwei Wang, Jiyu Zhang

**Affiliations:** 1 Lanzhou Institute of Husbandry and Pharmaceutical Sciences, Chinese Academy of Agricultural Sciences, Lanzhou 730050, China; lijiajing724@163.com (J.L.); chengfusheng@126.com (F.C.); weixiaojuan@caas.cn (X.W.); baiyb1011@163.com (Y.B.); qingwang880826@163.com (Q.W.); libing@caas.cn (B.L.); zhouyaxin@caas.cn (Y.Z.); zhaibingtao@caas.cn (B.Z.); zhouxuzheng@caas.cn (X.Z.); 2 College of Life Science and Food Engineering, Hebei University of Engineering, Congtai District, Handan 056038, China; 3 Key Laboratory of New Animal Drug Project of Gansu Province, Lanzhou 730050, China; 4 Key Laboratory of Veterinary Pharmaceutical Development, Ministry of Agriculture and Rural Affairs, Lanzhou 730050, China

**Keywords:** MRSA, epidemiology, resistance mechanisms, coping strategies

## Abstract

Increased antimicrobial resistance requires effective ways to overcome the global challenge of bacterial infections, including methicillin-resistant *Staphylococcus aureus* (MRSA). From the emergence of MRSA to its continued evolution, it is important to explore this pathogen from fresh perspectives and develop corresponding coping strategies to counter its growing threat. New coping strategies are continuously emerging, including but not limited to enhancing penetration capabilities or targeting their virulence. This review summarizes the epidemiological characteristics, drug resistance mechanisms, and therapeutic strategies of MRSA that have emerged over the past fifteen years. The focus of this paper is to explore the promising applications and current limitations of novel MRSA control strategies. This review serves as a key resource for treating MRSA infections and discussing novel strategies to overcome bacterial drug resistance.

## 1. Introduction

*Staphylococcus aureus* (*S. aureus*) has undergone natural selection [1] and clinical misuse of antibiotics [2,3], leading to the emergence of methicillin-resistant *Staphylococcus aureus* (MRSA). MRSA is commonly associated with respiratory, bloodstream, and skin infections, resulting in considerable morbidity and mortality [4]. In recent years, it has become one of the most prevalent bacteria in hospitals and the community. Also, MRSA has become a major pathogen in animal settings. The pathogen poses a great hazard to public health and livestock production [5,6,7]. Vancomycin has long been considered the most reliable drug to treat MRSA. However, despite its ability to inhibit MRSA activity in vitro, vancomycin has been reported in the literature as a therapeutic failure. The reason for this phenomenon is the emergence of hetero-resistant (hVISA) or glycopeptide-intermediate resistant S. aureus (VISA) [8,9]. This phenomenon is not exclusive to vancomycin but also occurs with gentamicin, oxacillin, daptomycin, and teicoplanin [10]. Alarmingly, this antibiotic also become ineffective due to the emergence of vancomycin-resistant *S. aureus* (VRSA) strains [11]. Therefore, medical and veterinary professionals must be aware of the potential dangers of MRSA infection and its varying levels of resistance to antibiotics in different countries. A concerted effort is needed to address the issue. This review discusses current MRSA prevalence and resistance mechanisms as well as coping treatment strategies for MRSA infection.

## 2. MRSA Types, Prevalence, and Spread

Over the past few decades, the prevalence and spread of MRSA has been constantly changing, with new MRSA clones appearing in different countries and regions. Therefore, it is necessary to monitor the characteristics, host specificity, and transmission routes of new strains and monitor continuous surveillance of MRSA in One Health settings [12,13]. From an epidemiological perspective, MRSA is commonly classified into three types based on their origin and prevalence. Each of these types has evolved in a variety of contexts and has its own unique genomic profile [14]. The three types of MRSA are healthcare-associated MRSA (HA-MRSA) [15], community-associated MRSA (CA-MRSA) [16,17], and livestock-associated MRSA (LA-MRSA) [18].

### 2.1. HA-MRSA

**Figure 1:**
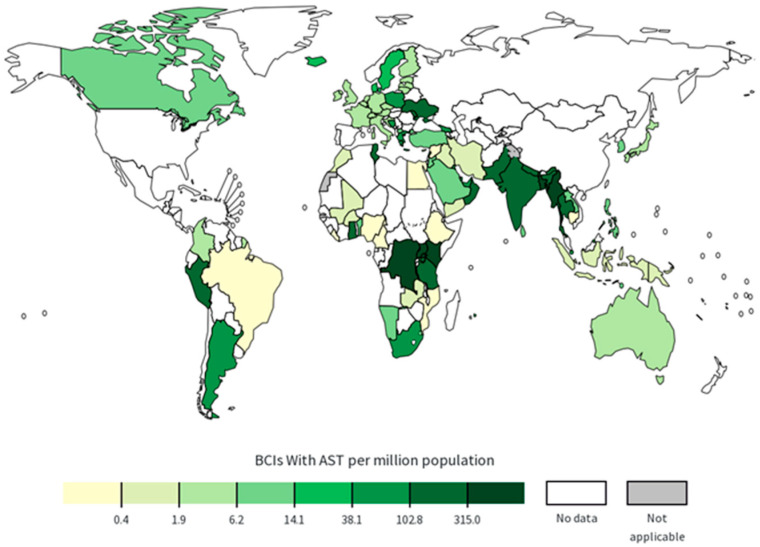
Global maps of confirmed bloodstream infections caused by MRSA in 2022 [26].

From its first discovery in 1961 until the 1980s, MRSA infections occurred mainly in hospitalized patients or those who had worked in a hospital setting and were termed HA-MRSA [19]. HA-MRSA remains one of the most common causes of multidrug-resistant nosocomial infections and remains a nosocomial pathogen with high incidence in many parts of the world [14,20]. HA-MRSA infections are often difficult to treat clinically because they are resistant to multiple antibiotics, resulting in high mortality and morbidity [20], suggesting that the prevalence and evolution of HA-MRSA cannot be overlooked. According to the World Health Organization (WHO) Global Antimicrobial Resistance and Use Surveillance System (GLASS), in 2022, East Asia and Africa were the regions with the highest number of reported bloodstream infections caused by MRSA among the countries and regions participating in the monitoring of GLASS (Figure 1). For sequence type (ST), previous studies have reported that the global pandemic clones of HA-MRSA mainly consist of two types, namely ST239 and ST5 [21,22]. In addition, another *S. aureus* strain known as epidemic MRSA-15 (EMRSA-15) belonging to ST22 is widely disseminated worldwide. In Europe, ST22 is the main HA-MRSA clone and spreads rapidly. In recent years, it has also been increasingly reported from Asia, including China, Russia, and India [23,24,25]. ST22 has the potential to spread globally and should be given high priority.

### 2.2. CA-MRSA

However, since the mid-1990s, there has been an increase in the number of reported MRSA infections in community populations with no exposure to healthcare facilities, and this increase has been associated with the recognition of new strains of MRSA, commonly referred to as CA-MRSA [27]. The emergence of CA-MRSA has fundamentally changed the epidemiological profile of MRSA isolates, with higher levels of virulence and rapid transmission, and has been one of the main reasons for the increased disease burden over the past decade [28]. CA-MRSA infection is defined as MRSA infection diagnosed in an outpatient clinic or within 48 h of hospitalization without hemodialysis, surgery, or the presence of an indwelling urinary catheter or percutaneous puncture device during a hospitalization more than one year earlier [29]. Compared with HA-MRSA, CA-MRSA is mainly associated with skin and soft tissue infections and is more resistant to β-lactams [30]. Differences in CA-MRSA strains were more pronounced in different geographic regions than other MRSA types. For example, in the Asia-Pacific region, the ST59 clone dominates, while in the United States, the dominant strain identified is ST8, which exhibits resistance to MRSA-sensitive clindamycin and trimethoprim-sulfamethoxazole (TMP-SMX) [31]. In contrast, ST80 is particularly prevalent in Europe [32].

### 2.3. LA-MRSA

The origin and transmission of LA-MRSA is mainly attributed to the irrational use of antibiotics in animal settings and close contact between humans and different animal species due to increased risk of animal exposure and transmission to humans [33]. Humans who are in close contact with animals are at an increased risk of colonization by LA-MRSA and subsequently infection with LA-MRSA [34]. Such cases of MRSA infections indicate its zoonotic transmission, thus posing a serious threat to the human population [35]. Studies have demonstrated that the probability of carrying MRSA in the most common companion animals (dogs and cats) was alarmingly high, and all *S. aureus* strains isolated from pets carried the *mecA* gene [36,37]. The number of pet owners has risen globally in recent years, and the transmission of MRSA from pets to owners can be devastating in the future. In France, MRSA was observed to colonize 39.3% of dogs, 26.5% of cats, and up to 47.1% of horses. Up to 72.1% of MRSA strains isolated from horses belonged to CC398 [38]. Another study conducted in Greece noted that the prevalence of *S. aureus* and methicillin-susceptible *S. aureus* (MSSA) were found to be 37% and 30%, respectively, while the overall prevalence of MRSA was 10.8% [39]. In European Union (EU) countries, the possible transmission of LA-MRSA clones was detected in clinical samples, suggesting that LA-MRSA clones can transmit between animals and humans. LA-MRSA strains have been detected in humans in 17 out of 19 EU countries, with the highest prevalence of LA-MRSA in the Netherlands (30.7%), Denmark (29.3%), and Spain (9.7%) [40]. In addition, this phenomenon has been observed in the United States [41], Canada [42], China [43], and India [44]. Therefore, close contact may be one of the potential ways for MRSA transmission to further persist, spread widely, and become difficult to control.

## 3. MRSA Resistance Mechanisms

The development of antimicrobial resistance (AMR) poses a great threat to global public health and economic stability. With the emergence of multi-drug resistance in MRSA, its resistance mechanisms have become more complex. This section explores the complex resistance mechanisms of MRSA, spanning genetic determinants to phenotypic adaptations. An in-depth study of the mechanisms of MRSA resistance can not only effectively demonstrate the evolution of MRSA but also contribute to the development of novel anti-infective drugs and control strategies.

### 3.1. Inherent Resistance Mechanisms

The core molecular basis of MRSA’s intrinsic resistance is the *mecA* gene, which is encoded by penicillin-binding protein 2a (PBP2a). This protein provides a robust defense barrier against *β*-lactam antibiotics by reconfiguring the cell wall synthesis pathway [45]. However, this classical mechanism does not fully account for the heterogeneity and environmental adaptation of resistant MRSA phenotypes—fluctuations in resistance profiles due to differences in staphylococcal chromosomal casette *mec* (SCC*mec*) type [40]. Recent studies have revealed that the *fem* gene family influences the PBP2a functional efficiency by regulating the peptidoglycan precursor metabolism [46,47], whereas the *agr* quorum-sensing system indirectly shapes resistance phenotypes (e.g., biofilm formation versus antibiotic osmotic resistance) by dynamically regulating virulence factor expression [48]. These findings suggest that intrinsic resistance to MRSA is essentially a complex system driven by core genes and supported by multi-level regulatory networks. Moreover, MRSA’s synergistic adaptation with acquired resistance mechanisms (e.g., activation of efflux pumps or target mutations) further exacerbates the clinical therapeutic challenges.

#### 3.1.1. SCCmec Element and PBP2a Protein

The SCC*mec* element is a mobile genetic element that carries *mecA* and its regulatory genes and can be readily transferred from one organism to another. Its insertion into the chromosomes of susceptible strains produces penicillin-binding proteins (PBP 2a/2c), which bind to *β*-lactam antibiotics with very low affinity, resulting in the inability of the drug to inhibit cell wall synthesis and thus developing resistance to *β*-lactams [49]. MSSA changes to MRSA upon the acquisition of staphylococcal cassette chromosome *mec* (SCC*mec*), a genomic island that encodes methicillin resistance [50]. The SCC*mec* element commonly ranges in size from 21 to 60 kb [51]. In addition to *mecA*, SCC*mec* can also carry other genetic elements, resulting in more diverse drug resistance or other biological functions. The basic structure of SCC*mec* can be divided into three parts: the *mec* gene complex, the *ccr* gene complex, and the junction region (J region). The classification of SCC*mec* is based on the *mec* and *ccr* gene complexes, with different combinations of *mec-ccr* gene complexes belonging to different classes of SCC*mec*, while sub-classes of SCC*mec* are based on the J-regions within the same class. Up to now, a total of 13 classes of SCC*mec* elements have been recognized, while many other reported SCC*mec* elements have not been recognized [12,52]. Among them, hospital-acquired MRSA (HA-MRSA) mostly carries SCC*mec* elements of type I, II, and III, while community-acquired MRSA (CA-MRSA) mostly carries type IV, which is more virulent but has a narrower resistance spectrum [50].

#### 3.1.2. β-Lactamase

The main reason for the high level *β*-lactam resistance by clinical isolates of MRSA is the presence of the *blaZ* gene that encodes for the production of a specific hydrolytic enzyme, *β*-lactamase, which hydrolyzes the *β*-lactam ring, resulting in inactivation of the drug [53]. The detailed mechanism is that the *blaZ* gene is regulated by the *blaR1-blaI* system. When the *blaR1* protein receives the stimulus of *β*-lactam antibiotics, it causes the hydrolysis of the inhibitory protein *blaI* to detach from the binding site, which leads to the expression of the *blaZ* gene and the production of *β*-lactamase, resulting in the inactivation of *β*-lactam antibiotics [54]. However, it is worth noting that β-lactamase alone does not confer resistance to methicillin or other β-lactamase-resistant β-lactams. MRSA strains need to express both β-lactamase and PBP2a to have resistance to β-lactams [55].

#### 3.1.3. Efflux Pumping System

The efflux pump is a membrane transporter protein present in the bacterial cell membrane, which eliminates the damaging effects of xenobiotics (e.g., antibiotics, disinfectants, toxins, etc.) on the cell by decreasing their concentration within the cell [56,57]. When antibiotic-sensitive strains are frequently exposed to antimicrobial drugs, the expression of efflux coding genes are activated, which results in a much greater ability to efflux drugs and consequently lead to antimicrobial resistance. Currently, the efflux pumps involved in MRSA multidrug resistance are mainly classified into two groups [58]: chromosome-associated pumps, such as *NorA*, *NorB*, *NorC*, *MdeA*, *SdrM*, *LmrS*, *MepA*, and *SepA*, and plasmid-associated, including *QacA*, *QacB*, *QacG*, *Qac H*, *QacJ*, and *Smr*. *NorA*, the first and most studied efflux pump identified in *S. aureus* exhibits resistance to hydrophilic fluoroquinolones (ciprofloxacin, moxifloxacin), while *NorB* and *NorC* exhibit resistance to hydrophobic fluoroquinolones (norfloxacin and sparfloxacin). Among plasmid-associated pumps, *QacA* is considered to be the most important exocytosis system of MRSA [59]. This active efflux system does not rely on ATP hydrolysis but rather on active transport through an electrochemical gradient formed by H^+^ on both sides of the cell membrane. The active exocytosis system in bacteria is a reversible process, i.e., H^+^ moves from the outside of the cell to the inside of the cell, whereas harmful intracellular substances, such as dyes and antimicrobial drugs, flow from the inside to the outside of the cell. Further studies have also demonstrated that the active efflux system plays an important role in antimicrobial resistance in MRSA [60]. Because of their central role in multidrug resistance, efflux pumps are considered promising targets for developing new therapeutic strategies to combat MRSA. In addition, upregulation of *mepA* gene (which is associated with the efflux pump) expression has been reported in the literature to be the main mechanism of heterogeneous resistance to ciprofloxacin in *S. aureus* GD18_SA_529 [61].

#### 3.1.4. Alterations in Cell Membrane Permeability

MRSA has the property of altering the permeability of its cell membrane. When the permeability of the cell membrane is reduced, MRSA can develop resistance to antimicrobial drugs by reducing the uptake of antimicrobials [62]. MRSA is able to reduce the uptake of aminoglycoside antibiotics through this property, which ultimately leads to a reduction in the uptake of the antibiotics and to the development of resistance [63]. Point mutations in cell membrane genes are also a mechanism for generating hetero-resistant S. aureus subpopulations [10].

#### 3.1.5. Alterations in Cell Wall Permeability

Cell walls, as a vital building block of bacteria, play an important role in maintaining the cell shape and making them resistant to osmotic pressure [64]. The cell wall of Gram-positive bacteria is thicker than that of Gram-negative bacteria, and its main component is peptidoglycan [65]. As a result, MRSA resistance differs because of its increased peptidoglycan synthesis and thick cell wall, which prevent glycopeptides from entering the cell. Interaction with peptidoglycan precursors results in low levels of resistance to glycopeptides. For example, VISA strains frequently exhibit a marked increase in cell wall thickness, which might contribute to glycopeptide resistance by amplifying stem peptide targets or preventing access of the antibiotic to the underlying cell wall targets [66,67,68]. hVISA strains display significantly thickened cell walls with reduced peptidoglycan cross-linking. This thickened matrix traps vancomycin molecules by presenting excess D-Ala-D-Ala termini, effectively sequestering the drug away from the lipid II precursors needed for cell wall synthesis. This mechanism reduces vancomycin’s ability to inhibit cell wall biosynthesis efficiently and is consistently observed across clinical hVISA isolates [69,70,71].

#### 3.1.6. Auxiliary Gene Regulation

In addition to *mecA* and *mecC*, there are a number of genes on the *S. aureus* chromosome that are also involved in the development of MRSA resistance. These genes are generally associated with peptidoglycan synthesis, cell division, and cell wall metabolism.

*fem* is a gene that is independent of *mecA* and *mecC*. Although the *fem* gene family is not directly involved in coding for PBP 2a, it plays a role as a “behind-the-scenes regulator” in maintaining intrinsic resistance to MRSA. This gene is involved in bacterial peptidoglycan synthesis, cell division, and cell wall metabolism, which can enhance MRSA resistance to *β*-lactam antibiotics [72,73]. *fem* genes encode proteins that catalyze the addition of glycine residues to the pentaglycine bridge of the peptidoglycan structure, a critical component for the proper cross-linking of the bacterial cell wall. FemX, FemA, and FemB participate in the synthesis of peptidoglycans in the cell wall by adding the first glycine unit, the second and third glycines, and the fourth and fifth glycines to the pentaglytic peptide bridge, respectively [47,74].

Auxiliary factors (Aux) provide necessary precursors and support for proper cell wall synthesis, which is critical for beta-lactam resistance [55]. They include genes involved in nitrogen metabolism, fatty acid biosynthesis, and modifications of the cell membrane lipids, among others. MRSA mutants lacking AuxA and AuxB have increased susceptibility to beta-lactam antibiotics, despite no changes in PBP2a (the *mecA* protein) expression, peptidoglycan cross-linking, or wall teichoic acid synthesis. This indicates these Aux proteins act through mechanisms other than altering PBP2a levels or major cell wall structural features [75].

### 3.2. Acquired Resistance Mechanisms

#### 3.2.1. Antibiotic Target Modification

Long-term antibiotic abuse can lead to the emergence of new resistance strains such as hetero-resistant *S. aureus* and vancomycin-resistant *S. aureus* (VRSA). In the human pathogen *S. aureus*, heterogeneous resistance is widespread and caused by common point mutations in core genes, including those involved in membrane and peptidoglycan/teichoic acid biosynthesis and transport, as a mechanisms for generating resistant subpopulations [10]. VRSA emerges by acquiring the *vanA* gene from other strains, usually from vancomycin-resistant enterococci (VRE). The *vanA* gene is located with the Tn1546 transposon, which is achieved by replacement of the D-Ala at the C-terminal of the peptidoglycan monomer pentapeptide with the vancomycin-low-affinity D-Lac or D-ser. The Tn1546 transposon was originally discovered in the spliced plasmid of vancomycin-resistant *Enterococcus faecalis*, which was transferred to *S. aureus* via horizontal gene transfer leading to VRSA strains [76]. Another mechanism of MRSA is resistance to linezolid, which most commonly happens due to point mutations in 23S Rrna at G2576T, T2500A, and G2447T positions, which make it difficult for linezolid to bind to its target site, leading to resistance [77,78].

#### 3.2.2. Biofilm Formation and Persistent Infection

Biofilm is a complex structure formed by bacteria under specific environmental conditions which is not inherent to the bacteria itself. Biofilm formation usually requires surface attachment, environmental signals (e.g., nutrient deprivation, antibiotic stress, pH change, etc.), Quorum Sensing (QS), and secretion of extracellular polymers (EPs). The molecular differences in biofilm composition between MSSA and MRSA primarily involve the types of biofilm matrix components and the genetic regulation underlying their production. MSSA biofilms are largely dependent on polysaccharides, specifically polysaccharide intercellular adhesin (PIA), which is produced by the *icaADBC* operon. MSSA biofilms tend to have higher polysaccharide content and are induced under osmotic stress conditions such as high NaCl concentrations [79]. MRSA biofilms, in contrast, are more proteinaceous and contain higher levels of extracellular DNA (eDNA). They rely less on PIA and the *ica* operon for biofilm formation. Instead, MRSA biofilms depend on surface proteins with LPXTG motifs and proteins encoded by serine-aspartate repeat (SDR) genes such as *sdrC* and *sdrD*, which are important for biofilm structure [80,81]. These differences create MRSA-produced biofilms with the ability to be more adaptive to the environment, evade immune cells phagocytosis, and exhibit drug resistance [82].

In addition, biofilms contain extensive dormant resident helper cells, such as persister cells, which enable the bacteria to maintain a low metabolic level, thus protecting them from antimicrobial damage and development of resistance to antimicrobials. This dormant state makes it difficult for ATP-dependent antibiotics to work properly to kill this bacteria [83]. Upon exiting the antimicrobial environment, biofilm-embedded cells can regain their growth capacity and restore infectivity.

## 4. MRSA Control Strategies

MRSA is resistant to penicillin, aminoglycosides, tetracyclines, macrolides, quinolones, and other drugs, and its resistance mechanism involves gene mutation, biofilm effect, drug efflux pumps, etc., which brings great challenges to the treatment of MRSA infections [84]. Currently, effective approaches for controlling MRSA infection include conventional antibiotics and novel control strategies. Vancomycin is undoubtedly the drug of choice for the clinical treatment of MRSA, but in recent years, due to the irrational use of vancomycin, the sensitivity of MRSA to vancomycin has been decreasing. Moreover, the clinical efficacy is unsatisfactory, and ototoxicity and nephrotoxicity are caused by high-dose treatment. Even vancomycin-resistant *S. aureus* (VRSA) strains have appeared. Recently, the United States Food and Drug Administration (FDA) approved five antibiotics to treat MRSA infections, including linezolid, daptomycin [85], tigecycline, telavancin, and ceftaroline [86]. However, these antibiotics are becoming less effective due to mutations in the 23S rRNA gene [78].

**Figure 2:**
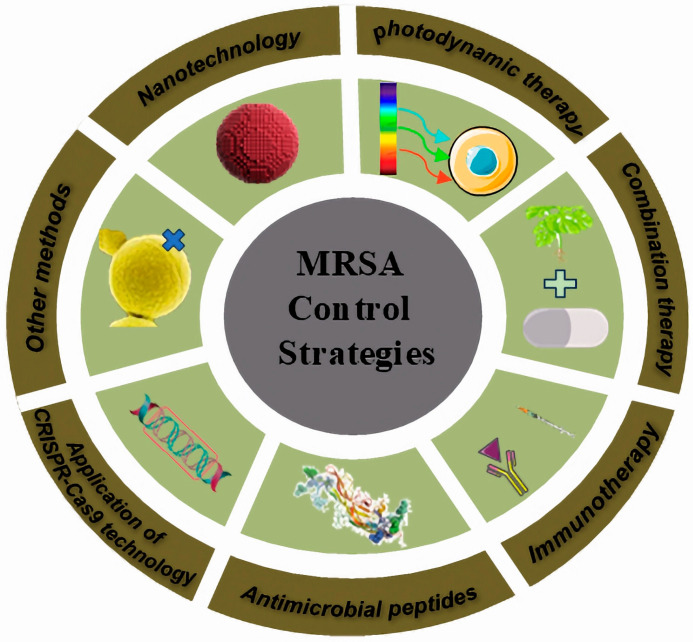
Summary of new control strategies for the treatment of MRSA infections.

With the exploration of novel antimicrobial materials, antimicrobial mechanisms, and antimicrobial targets, the research on novel control strategies for the treatment of MRSA infections has been diversifying. The novel control strategies described in this review mainly focus on six aspects: nanotechnology, photodynamic therapy, combination therapy, immunotherapy, antimicrobial peptides, and application of CRISPR-Cas9 technology (Figure 2).

### 4.1. Nanotechnology

In recent years, nanomaterials have been found to have unique optical, electrical, magnetic, chemical, and biological properties [87], which can overcome the existing MRSA resistance mechanisms, including inhibition of bacterial biofilm formation, increased uptake and decreased efflux of antimicrobial drugs, simultaneous loading of multiple antibiotics, and administration of higher doses of antimicrobial drugs targeting the site of infection [88]. On the one hand, a part of functional nanomaterials themselves can induce antimicrobial activity and reduce drug resistance [89]. The nanomaterials used as antimicrobial agents include metallic gold, silver, copper, zinc and its oxide nanomaterials, and carbon nanomaterials such as graphene oxide [90,91]. Most of the nanomaterials in this category cause bacterial death through targeted virulence mechanisms and inhibition of biofilm formation [92], cell membrane disruption [93], and other mechanisms, providing a promising alternative to traditional antibiotics. On the other hand, the unique structure of nanomaterials can be an efficient antimicrobial drug delivery carrier to achieve the unique properties of enhanced therapeutic effect, prolonged drug action time, and improved serum stability [94]. Liposomes, polymeric nanomaterials, metal and metal oxide nanomaterials, and carbon-based nanomaterials fall into this category. In addition, some nanomaterials combine antimicrobial activity and transport carrier properties to significantly increase their efficacy against MRSA infection [91,95]. Although nanotechnology provides innovative strategies against MRSA through enhanced drug delivery and antimicrobial activity, limitations such as targeting accuracy, stability, toxicity, biofilm penetration, and clinical translation remain significant barriers to widespread therapeutic applications [94,95,96,97].

### 4.2. Photodynamic Therapy

It is evident that photodynamic therapy (PDT) has recently garnered significant attention due to its noteworthy efficacy in eradicating a wide range of microorganisms, including MRSA [98]. Photodynamic therapy is a treatment that exerts biological effects through a combination of photo physical and photochemical processes and is an emerging non-invasive treatment that involves the use of light, a photosensitizer (PS), and endogenous molecular oxygen to kill microbial cells. It works by photoexcitation via a PS inducing a photochemical reaction with the cellular substrate or molecular oxygen to generate reactive oxygen species (ROS), which promotes cellular oxidative stress, leading to DNA damage, alteration in cell membrane permeability, destruction of proteins, and other components, ultimately leading to cell death [99]. In its early stages of development, PDT was used as a therapeutic modality for the treatment of malignant tumors. Subsequent advancements in the technology led to the emergence of PDT as a treatment for bacterial infections, which is now referred to as antimicrobial photodynamic therapy (aPDT). The therapeutic principle of aDPT is that ROS produced by photosensitizer-mediated aPDT, such as 5-Aminolevulinic acid [100] and mono substituted tricationic Zn (II) phthalocyanine [101], cause lethal injury to bacteria by damaging DNA and altering the permeability of the cytoplasmic membrane. In the presence of a PS and an appropriate wavelength of light, the PS absorbs light and initiates the formation of toxic substances. Moreover, it has been shown that aPDT in combination with antibiotics achieves an additive effect in eliminating drug resistance in MRSA strains. A study conducted by Willis et al. [102] has shown that aPDT with the PS methylene blue in combination with chloramphenicol and tetracycline significantly reduced resistance to two MRSA strains (USA300 and RN4220). Recently, it was found that only 5 μg/mL of Brazilian green propolis was used as PS to enhance the bactericidal effect against MRSA (ATCC 43300) after activation by high-intensity blue LED light. This suggests that Brazilian green propolis has great potential as an aPDT photosensitizer, and this method is expected to be an effective treatment for MRSA in clinical practice [103]. Additionally, studies have examined three aggregation-induced emission luminogens (AIEgen), namely, TI, TBI, and TTI, as PSs to control MRSA infections. The final study showed that, except for TBI, both TI and TTI could have strong inhibitory effects on MRSA. Even in the absence of light, TI inactivated 90% of MRSA within 30 min. TTI not only killed more than 99% of MRSA but also limited intracellular MRSA survival by upregulating the immune response of macrophages [104]. In summary, PDT offers a promising alternative or adjunctive approach for MRSA skin infections, but its limitations include photo bleaching of photosensitizers, restricted light penetration, potential host tissue damage, variable strain susceptibility, requirement for repeated treatments, and limited clinical validation to date [105,106]. Notably, photo bleaching of photosensitizers is both a critical factor in PDT and reduces the efficiency of bacterial inactivation [107].

### 4.3. Combination Therapy

Plant extracts are the active ingredients obtained from purely natural plants by a wide range of physical and chemical methods and consist primarily of polysaccharides, alkaloids, flavonoids, phenols, terpenoids, etc., which are widely distributed in nature and have antimicrobial properties. The combination of plant extracts and antibiotics in the treatment of MRSA not only significantly improves the antibacterial effect of antibiotics but also reduces the dosage of antibiotics and toxic side effects [108,109]. Combined administration of plant extracts can effectively affect the synthesis of efflux pumps, cellular structures (e.g., cellular membranes, cellular walls), and other resistance mechanisms of MRSA (Table 1). Chrysoeriol from *Artemisia rupestris* enhanced the inhibitory effects of norfloxacin, ciprofloxacin, and oxacillin against MRSA of clinical origin. Chrysoeriol in combination with ciprofloxacin showed a strong synergistic effect on the fractional inhibitory concentration index (FICI) of SA1199B (<0.024), with a 128-fold reduction in the dose of ciprofloxacin. The FICI of chrysoeriol and ciprofloxacin against SA1199B was less than 0.024, showing strong synergistic effects and reducing the use of ciprofloxacin by 128 times. These results showed that artemisinin flavonoids could effectively reduce the resistance of MRSA to antibiotics [110]. The synergistic effect produced by the two compounds significantly reduced the amount of mRNA, which led to a decrease in the synthesis of the NorA efflux pump and inhibited drug efflux in MRSA strain SA1199B. Clerodane diterpene 16 α-hydroxycleroda-3,13(14)- Z -dien-15,16-olide (CD) extracted from the leaves of *Polyathia longifolia* enhanced the inhibitory effects of tetracycline, daptomycin, and linezolid on MRSA clinical isolates. Nine of them showed a strong synergistic effect, which could reduce the dosage of norfloxacin by 4–16 times, and the remaining six strains showed additive interaction (FICI = 0.624–0.750). In combination with ciprofloxacin, CD enhanced the antibacterial activity of ciprofloxacin against six clinical MRSA isolates by four to eight times (FICI = 0.324). The combination of CD with oxfloxacin showed a synergistic effect of four to eight times on the FICI of eight MRSA clinical isolates (FICI = 0.324). These results showed that terpenoids can effectively reduce the antibiotic resistance of MRSA [111].

### 4.4. Immunotherapy

One of the current therapeutic strategies to target the immune escape mechanism of MRSA is active immunotherapy using vaccine-based agents and passive immunotherapy using antibody-based agents to neutralize virulence factors or bind to the pathogens to promote phagocytosis.

Vaccines such as V710 [120], SA3Ag and SA4Ag [14,121,122], GSK2392102A [123], and rAT [124] are active immunotherapeutic drugs developed by several pharmaceutical companies such as Merck and Pfizer. Most of the vaccines have adverse reactions leading to termination of clinical studies of the vaccines due to poor clinical efficacy and poor safety. So far, vaccine development for MRSA has been mostly unsatisfactory, and there is no vaccine against *S. aureus* for the control of MRSA infection. On a positive note, however, the LTB-SA7 vaccine developed by LimmaTech Biologics is currently in Phase I clinical trials in the United States [125].

Passive immunotherapy is mainly based on monoclonal antibodies against different pathogenic factors, peptide immune modulators, and antisense gene therapy. Among monoclonal antibodies, hUK-66 IgG1 [126], pagibaximab [127], and the α-hemolysin antibody [128] reduced the mortality of humans from MRSA infection. However, passive immunotherapy has not been used for clinical treatment so far.

### 4.5. Antimicrobial Peptides

Antimicrobial peptides (AMPs), also known as host defense peptides (HDPs), are small molecules produced by the natural immune system of the host. Most antimicrobial peptides exhibit broad-spectrum activity against diverse microorganisms, including bacteria, fungi, and viruses [129]. They are considered antimicrobial agents and have gained increasing attention as an alternative to antibiotic therapy due to several advantages, such as high antimicrobial efficiency and broad spectrum of antimicrobial activity, as well as being less likely to result in the development of resistance. While AMPs offer a novel mechanism to combat antibiotic-resistant bacteria, its clinical application has been hindered by variable efficacy, complex host–pathogen interactions, delivery challenges, and the need for personalized or optimized treatment protocols [130].

For example, the antimicrobial peptide GN1 [131] inhibited the transcription of the drug-resistant gene *mecA* and bound to PBP2a, thus synergistically blocking the synthesis of the cell wall of drug-resistant bacteria with *β*-lactam antibiotics. GN1 antagonized the activity of *β*-lactamase and prevented the hydrolysis of antibiotics. In addition, GN1 inhibits the synthesis of MRSA biofilms and staphyloxanthins, making them more susceptible to oxidative damage. Eventually, GN1 interferes with bacterial metabolism by inducing the production of large amounts of ROS and promoting the intracellular accumulation of glutamic acid in the cell, leading to bacterial cell death. In another study by Awdhesh et al. [132], AM1 inhibited the growth of MRSA at lower antibiotic MIC concentrations by disrupting bacterial cell membrane integrity and inhibiting the formation of MRSA biofilms.

### 4.6. Application of CRISPR-Cas9 Technology

The CRISPR-Cas9 system has more delivery methods, including physical, chemical, and biological methods [133]. Physical methods require creating a transient gap in the phospholipid bilayer of the cell membrane or injection directly into the cell, including via electroporation, microinjection, hydrodynamic injection, membrane deformation, ultrasound, laser irradiation, and osmosis. Physical methods are easy to manipulate but damage cells and cause irreversible physiological damage to cell membranes [133]. Chemical methods are flexible and versatile by chemically modifying the complexes and then delivering them into cells, for example, after inorganic solvent processing or by assembling Cas9-sgRNA complexes with organic or composite materials. Biological methods include phage, virus, and extracellular vesicle delivery, among others. Thus, biological delivery methods are currently the most common delivery methods [133].

Ates et al. [134] constructed antimicrobial drugs based on CRISPR-Cas technology targeting methicillin (*mecA*), gentamicin (*aacA*), and ciprofloxacin (*grlA*, *grlB*) resistance genes to counteract multi-drug resistance in MRSA. They used a physical approach to electroporate CRISPR plasmids with specific single-guide RNAs into MRSA strains. Changes in gene expression and resistance status were assessed by real-time polymerase chain reaction (RT-PCR) and antibiotic susceptibility testing (AST), respectively. Surprisingly, AST demonstrated sensitivity to *β*-lactam, quinolone, and aminoglycoside antibiotics.

Shimamori et al. [135] efficiently synthesized CRISPR-Cas13a-antimicrobial capsids against MRSA using CRISPR-Cas13a technology for targeted and silent mutation elimination of MRSA strains carrying antibiotic resistance genes. The authors created CRISPR-Cas13a-loaded non-replicating phage particles (AB-capsids) designed to specifically recognize the *mecA* gene. CRISPR-Cas13a was delivered to MRSA cells, where it induced side-branch cleavage of RNA, effectively disrupting essential bacterial functions and causing cell death. This strategy demonstrates a promising approach to overcoming antimicrobial resistance through precise genetic targeting.

### 4.7. Other Methods

Other approaches includes application inhibitors of cell wall teichoic acid (WTA) synthesis [136], such as tunicamycin [137], which act synergistically with *β*-lactam antibiotics in the treatment of MRSA infections by destabilizing PBP2a. WTA, a phosphatidic ribitol polymer, is a major component of the cell wall of *S. aureus* [136]. Because PBP2a relies on glycosylated WTA as a scaffold, it is sensitive to *β*-lactam antibiotics when MRSA lacks WTA.

## 5. Conclusions and Future Prospects

The most common LA-MRSA CC398 accounts for only a small portion of human MRSA, but its high pathogenicity cannot be ignored. *MecC*-MRSA is distributed in all animal species, has a preference for animal hosts, and poses no specific threat to humans [138].

MRSA strains are becoming increasingly resistant to existing commonly used antibiotics, posing a significant threat to human and animal life, and improved antimicrobial drugs and strategies are urgently needed to combat bacterial resistance. New anti-MRSA drugs such as linezolid, daptomycin, tigecycline, and telavancin are also decreasing their effectivity in controlling MRSA due to the emergence of new resistance mechanisms such mutations in the 23S rRNA gene. The new MRSA control strategies such as nanotechnology, photodynamic therapy, immunotherapy, vaccine development, phage therapy, and CRISPR technologies provided better evidence-based insights to control the growing MRSA resistance. Researchers have also been actively developing the use of active plant extract ingredients in combination with antibiotics to break through the MRSA resistance. These in vitro combined applications highlight the clinical use of valuable active ingredients and existing antibiotics to treat drug-resistant pathogens. While novel strategies have broadened the field of ideas for treating MRSA, they still have a long way to go before they can be applied in clinics and require intensive research. If these new methods are used, there are questions about whether they will be reliably effective, stable, safe, and affordable in their application. In the future, as the research continues to deepen and progress, these approaches may provide a wider choice of strategies for the prevention and control of drug-resistant bacteria.

Although there are many options for the treatment of MRSA infection, and new antimicrobial drugs and therapeutic measures are constantly emerging, the fundamental strategy to fight against MRSA is to reduce the unjustified use of antimicrobial drugs to minimize the emergence of new bacterial drug-resistant strains and to control their further spread. MRSA spread can effectively be prevented by adopting behaviors such as washing hands frequently, keeping wounds clean, avoiding sharing personal items, and maintaining environmental hygiene. Hospitals can isolate MRSA-infected patients in single-occupancy wards, and healthcare workers should strictly enforce sterilization and protective measures when entering and leaving the wards. Avoid participating in high-risk activities when wounds cannot be properly dressed. Schools and other places can strengthen environmental cleaning and disinfection as well as present awareness seminars on MRSA knowledge and preventive measures. At the societal level, the state should develop and promote MRSA prevention and control guidelines and coordinate with institutions to implement infection control measures. Popularize knowledge about MRSA through the media and community activities and promote good hygiene practices to reduce community transmission. Ensure that healthcare institutions and communities have adequate protection resources and capacity, including testing isolation and treatment facilities.

## Figures and Tables

**Table 1:** Summary of plant extracts with anti-MRSA activity.

Structure Type	Plant Extracts	Antibiotics	FIC Index	MRSA Source (Strains Tested)	Mechanism of Action	References
Phenolic compounds	Lupinifolin	Ampicillin	<0.5625	DMST 20645	Cell wall, cell membrane	[112]
		Cloxacillin	<5156			
	Chrysoeriol	Norfloxacin	<0.266	SA1199B	NorA efflux pump	[110]
		Ciprofloxacin	<0.024			
		Oxacillin	<0.375			
	Epigallocatechin gallate	Tetracycline	0.5	Clinical MRSA (C1, ATCC 43300)	Cell membrane, β-lactamase, biofilm, virulence factors	[113]
		Mupirocin	0.5			
		Fusidic Acid	0.5			
	Ethyl gallate	Tetracycline	0.5			
		Mupirocin	0.5			
		Fusidic Acid	0.5			
	Theaflavin	Ceftiofur	0.1875	MRSA (USA300)	Cell wall	[114]
		Cefoxitin	0.3125			
		Latamoxef	0.3125			
		Ceftazidime	0.3125			
		Oxacillin	0.3125			
		Ampicillin	0.3125			
	Curcumin	Oxacillin		Clinical MRSA (7 strains)	Cell membrane, biofilm,	[115]
Alkaloids	Berberine	Gentamicin	0.53–1.06	Clinical MRSA (50 strains)	Membrane permeability, cell wall	[4]
		Levofloxacin	0.62–1.5			
		Amikacin	0.16–1.25			
	Chelerythrine	Oxacillin	0.5	MRSA (ATCC 43300, ATCC 700699)	Cell wall, cell membrane	[116]
Terpenoids	Clerodane diterpene 16 α-hydroxycleroda-3,13(14)-Z-dien-15,16-olide	Norfloxacin	0.315–0.5	Clinical MRSA (9 strains)	Efflux pump	[111]
		Oxfloxacin	0.324	Clinical MRSA (8 strains)		
		Ciprofloxacin	0.324	Clinical MRSA (6 strains)		
	Ursolic acid	Tetracycline	0.28	MRSA (ATCC 43300)	Cell wall, PBP2a protein, β-lactamase	[117]
	Cleanolic acid	Oxacillin	0.5	MRSA (ATCC 43300)	Cell wall, PBP2a protein, β-lactamase	[117]
Essential oils	Thymol	Vancomycin	1.0	MRSA (TCH1516)	Biofilm	[118]
	Carvacrol	Cefotaxime	0.28–0.5	MRSA (SA-70, SA-372, 161402)	Biofilm	[119]
		Oxacillin	0.28–0.5			
		Ampicillin	0.375–0.5			

## Data Availability

No new data were created or analyzed in this study. Data sharing is not applicable to this article.
